# Marine Glycoconjugates: Trends and Perspectives

**DOI:** 10.3390/md18020120

**Published:** 2020-02-18

**Authors:** Vladimir I. Kalinin, Valentin A. Stonik, Natalia V. Ivanchina

**Affiliations:** G.B. Elyakov Pacific Institute of Bioorganic Chemistry, Far Eastern Branch of the Russian Academy of Sciences, Pr. 100-letya Vladivostoka 159, 690022 Vladivostok, Russia; stonik@piboc.dvo.ru (V.A.S.); ivanchina@piboc.dvo.ru (N.V.I.)

Glycoconjugates play significant roles in biological systems and are used in medicine, for example as vaccines. Glycoproteins, peptidoglycans, lipopolysaccharides, and other biopolymer glycoconjugates are responsible for cellular interactions, including cell–cell recognition and the binding of cells to the intercellular matrix. These molecules perform signal, antigenic and transport functions, and participate in the formation of receptors and other important membrane and blood constituents. Due to the negative charges of some sulfated glycoconjugates and the binding of water, they are critical for maintaining the physical status of connective tissue [1]. Low molecular weight glycoconjugates, such as triterpene and steroidal glycosides [2,3,4], glycolipids, are also well known as molecules playing important internal and exterior roles. Diverse glycoconjugates show a very wide spectrum of biological activities, including defensive, cytotoxic, antitumor, immunomodulatory, and antioxidant properties. The water environment requires high solubility for signal or anti-predatory exometabolites of marine organisms, and carbohydrate moieties provide this property. All these peculiarities explain the very wide diversity of glycoconjugates of marine origin, including those presented in the current Special Issue.

Sterol and sphingolipid glycoconjugates, which are widespread, but insufficiently studied metabolites of microalgae, were discussed by Stonik and Stonik in the review article. Glycosylated sterols play important biological roles in microalgae and show different beneficial properties useful for medicine and healthy food. Dietary sterols and their glycoconjugates of microalgae enter into marine invertebrates through food chains and may be converted into 7(8)-unsaturated sterols and their derivatives, such as polyhydroxylated sterols and, probably, glycosides of starfishes and sea cucumbers. The knowledge of microalgal glycosphingolipids still remains poor, despite intensive investigations. Some of them are important for their interactions with pathogens and may induce apoptosis in microalgae. They also participate in the termination of microalgal blooms [5].

In their experimental article, Galasso et al. have discussed the obtaining and properties of a water-soluble bioactive fraction isolated from the toxic dinoflagellate *Alexandrium minutem.* This substance is a glycoprotein with a molecular weight above 20 kDa. It demonstrates specific antiproliferative activity (IC_50_ = 0.4 µg/mL) against the A549 cell line (the human lung adenocarcinoma cells). Moreover, the glycoconjugate did not reveal a cytotoxicity against human normal lung fibroblasts (WI38), but induced cell death, triggered by mitochondrial autophagy (mitophagy) in tumor cells. No mitophagic events were activated by it in normal WI38 cells [6].

Kostetsky et al. from the Far East Federal University (Vladivostok, Russia) have compared the fatty acid composition and thermal transitions of membrane lipids from green macroalgae *Ulva lactuca,* collected in the Sea of Japan and the Adriatic Sea. The adaptation to a warmer climatic zone was accompanied by a significant decrease in the ratio between unsaturated and saturated fatty acids (UFA/SFA) in membrane lipids. The decreasing ratio n-3/n-6 polyunsaturated fatty acids (PUFAs) was found in extra-plastidial lipids and only in the major glycolipid, non-lamellar monogalactosyldiacylglycerol. The opposite thermotropic behavior of non-lamellar and lamellar glycolipids can contribute to the maintenance of the highly dynamic structure of thylakoid membranes [7].

The role of marine and freshwater lectins, compounds specifically recognizing carbohydrate ligands, as anticancer agents, has been discussed by Italian and American scientists in the review “Antitumor Potential of Marine and Freshwater Lectins,” of Catanazaro et al. The co-authors have concluded that lectins from aquatic organism, demonstrating a great variety of inhibitory effects against tumor cells and triggering apoptosis and other forms of cell death, promise a bright future for such lectins in anticancer research. Some these lectins are able to enhance the antineoplastic activity of common antitumor medications. In the majority of cases, the lectins can distinguish normal and transformed cells and even different types of tumor cells due to their ability to recognize glycosylated patterns of various cell types. The lectins tested on animals revealed optimal antitumor activities at negligible toxicity [8].

In their experimental work, Chinese scientists (Tao Wu et al.) from Zhejiang Sci-Tech University, Hangzhou, have reported that a gene encoding marine sponge *Aphrocallistes vastus* C-type lectin (AVL) was inserted into an oncolytic vaccinia virus vector (oncoVV) to form a recombinant virus oncoVV-AVL. In vivo experiments showed that oncoVV-AVL induces a significant antitumor effect in colorectal cancer and liver cancer mouse models. These findings open the possibility of using the virus, containing marine lectin AVL, in oncolytic viral therapies [9].

One more lectin, OXYL, a type-2 LacNAc-binding 14 kDa lectin belonging to the C1qDC family, was isolated and characterized from the feather star *Anneissia japonica* (Echinodermata, Crinoidea) by an international team (Hasan, et al.) led by Professor Yasuhiro Ozeki (Japan). The structural studies of this lectin, carried out by Edman degradation, revealed its N-terminal region, including the first 40 amino acids of the mature protein. Its functional characteristics have been studied in detail. The lectin function relates to immunity in the organism-producer. It caused a strong aggregation of bacterial cells, but did not act on their growth. The lectin also displayed remarkable LacNAc recognition-dependent anti-biofilm activity. The authors supposed that, due to its novel primary structure and unique activities, this lectin may be estimated as a living fossil, revealing the structural and functional diversification of metazoan lectins [10].

New low-molecular-weight glycoconjugated compounds of echinoderms were isolated from different species and studied by scientists from G.B. Elyakov Pacific Institute of Bioorganic Chemistry, PIBOC (Russian Federation). For example, three new steroidal glycosides, anthenosides V–X, along with seven previously known anthenosides, E, G, J, K, S_1_, S_4_, and S_6_, have been isolated from the extract of tropical starfish *Anthenea aspera* by Malyarenko et al. It is of interest that anthenoside V contains a rare 5α-cholest-8(14)-en-3α,7β,16α-trihydroxysteroidal nucleus. All the investigated compounds at nontoxic concentrations inhibit the colony formation of human melanoma RPMI-7951, breast cancer T-47D, and colorectal carcinoma HT-29 cell lines. The mixture of anthenosides J and K possesses significant anticancer activity and induces apoptosis of HT-29 cells. The mechanism of the proapoptotic action of this mixture was found to be associated with the regulation of anti- and proapoptotic protein expression, followed by the activation of initiator and effector caspases [11].

Metabolomic studies concerning the localization of polar steroids, including their glycoconjugates, in different body components of the Far Eastern starfish *Lethasterias fusca,* have been carried out using nanoflow liquid chromatography/mass spectrometry with captive spray ionization (nLC/CSI–QTOF–MS) by Popov et al. The assumptions concerning the digestive function of polyhydroxysteroids and their derivatives and the protective role of asterosaponins in starfish were in good agreement with the obtained results. The highest level of polar steroids and their glycoconjugates was found in the stomach. Asterosaponins were found in all other body components; the main share of free polyhydroxysteroids and related glycosides were located in the pyloric caeca [12].

Seven new sulfated triterpene glycosides, psolusosides B, E, F, G, H, H_1_, and I, and the earlier-known psolusoside A and colochiroside D, have been isolated by Sichenko et al. from the sea cucumber *Psolus fabricii,* collected in the Sea of Okhotsk. The structure of psolusoside B has been revised using modern 2D-NMR and HR-ESIMS procedures and re-established as a disulfated tetraoside. The structures of other glycosides were elucidated in the same manner. Cytotoxic activities of psolusosides E, F, G, H, H_1_, and Iagainst the mouse ascite Ehrlich carcinoma cells, erythrocytes, neuroblastoma Neuro 2A and normal epithelial JB-6 cells were quite different, while the hemolytic action of the tested compounds was higher than their cytotoxicity against other cells, particularly against the Ehrlich ascites carcinoma. Psolusoside G was not cytotoxic against normal JB-6 cells, but revealed high activity against Neuro 2A cells. The cytotoxic activity against human colorectal adenocarcinoma HT-29 cells and the influence on the colony formation and growth of HT-29 cells by psolusosides B, E, F, H, H_1_, and I, along with psolusoside A as a control, were examined. The highest inhibitory activities were shown for psolusosides E and F [13].

Additionally, 10 new di-, tri- and tetrasulfated triterpene glycosides, psolusosides B_1_, B_2_, J, K, L, M, N, O, P, and Q, have been also isolated from the sea cucumber *Psolus fabricii* by the same team. The cytotoxic activities of these psolusosides on several mouse cell lines, including Ehrlich ascites carcinoma cells, neuroblastoma Neuro 2A, normal epithelial JB-6 cells, and erythrocytes, were quite different depending both on the structural peculiarities of these glycosides and the type of cells. The most interesting finding was that psolusosides P and Q contain four sulfate groups in their carbohydrate chains. [14]. The presence of four sulfate groups is extremely rare in low molecular weight metabolites. However, examples of substances having four and even more sulfate groups were earlier reported in the studies on another class of metabolites, namely on polysulfated sterol dimers, hamigerols A and B, from the Mediterranean sponge *Hamigera hamigera* [15]. Moreover, axinelloside A, an unprecedented, highly sulfated lipooligosaccharide from the marine sponge *Axinella infundibula*, contains 19 sufate groups attached to 12 sugars [16]. Nevertheless, the finding of tetrasulfated derivative is also unique, particularly in relation to echinoderm metabolites.

Yunmei Chen et al. from Gingdao Ocean University of China have discussed an in vivo affect of glycosaminoglycan AHG from the edible sea cucumber *Apostichopus japonicus* on hyperglycemia in the liver of insulin-resistant mice. The obtained results demonstrated that AHG supplementation significantly decreased body weight, level of blood glucose and content of serum insulin in a dose-dependent manner in the mice. The protein levels and gene expression of gluconeogenesis rate-limiting enzymes G6Pase and PEPCK were significantly decreased. Although the total expression of IRS1, Akt, and AMPK in the insulin-resistant liver was not affected by AHG supplementation, the phosphorylations of IRS1, Akt, and AMPK were clearly increased by AHG treatment. The authors of this article concluded that AHG could be a promising candidate for the development of an antihyperglycemic agent [17].

Several reports in this Issue concern the bioactive compounds of microorganisms. Zhuravleva et al. from PIBOC (Vladivostok, Russian Federation) have described in their article 10 new diterpene glycosides, virescenosides Z9–Z18 isolated from a marine strain of the fungus *Acremonium striatisporum* KMM 4401 associated with the sea cucumber *Eupentacta fraudatrix*. Glycosides of this class bear rare monosaccharide units such as altrose. Moreover, virescenosides Z12–Z16 are monosides containing unique methyl esters of altruronic acid. The carbohydrate moiety of virescenoside Z18 was found to be a methylester of mannuronic acid. The effects of some glycosides and aglycons on urease activity and the regulation of ROS and NO production in murine macrophages, stimulated with lipopolysaccharide (LPC), were also studied [18].

A lipopolysaccharide (LPS), including the O-specific polysaccharide (O-antigen) of *Aeromonas veronii* bv. *sobria* strain K557, serogroup O6, isolated from the carp *Cyprinus carpio* during an outbreak of motile aeromonad septicemia (MAS) on a Polish fish farm, has been immunochemically studied by Dworaczek et al. Freshwater *C. carpio* is a common inhabitant of the bays and lagoons of Southern part of the Baltic Sea. The O-polysaccharide was obtained by acid hydrolysis of the LPS and studied by chemical transformations and spectral methods including ^1^H and ^13^C NMR spectroscopy. The O-antigen comprises two O-polysaccharides, both containing a unique sugar, 4-amino-4,6-dideoxy-l-mannose (N-acetyl-l-perosamine, l-Rhap4NAc). Western blotting and an enzyme-linked immunosorbent assay (ELISA) revealed that the cross-reactivity between the LPS of *A. veronii* bv. *Sobria* K557 and the *A. hydrophila* JCM3968 O6 antiserum, and vice versa, is caused by the occurrence of common disaccharides, whereas an additional →4)-α-D-GalpNAc-associated epitope defines the specificity of the O6 reference antiserum. This investigation provides additional knowledge of the immunospecificity of *Aeromonas* bacteria and seems to be significant for epidemiological studies and for finding the routes of transmission of pathogenicity [19].

The diversity of marine glycoconjugate sources and their chemical structures, described in this Special Issue, is impressive. Two articles concern microalgal metabolites such as steroid and sphingoid glycoconjugates, and a glycoprotein from a sea cucumber with interesting biological activities, respectively [5,6]. One article discusses the fatty acid composition and thermotropic behavior of glycolipids and other membrane lipids of green macrophyte *Ulva lactuca* [7]. Three articles cover lectin subjects [8,9,10]. One review article analyzes the results and perspectives of marine and freshwater lectins’ application in experimental oncology and the therapy of oncological diseases; another article describes the use of a sponge lectin in the construction of a recombinant virus. The third article concerns the function of the immunity of a lectin in producing this compound crinoid. Two articles concern steroid glycosides from starfish [11,12], and two others concern triterpene glycosides from sea cucumbers [13,14]. One article describes the effect of a glycosaminoglycan from the sea cucumber *Apostichopus japonicus* on hyperglycemia in the liver of insulin-resistant mice [17]. One article concerns the isolation of 10 new triterpene glycosides from a fungus associated with a sea cucumber [18]. The article by Dworaczek et al. characterizes the O-specific polysaccharide (O-antigen) of a bacterial pathogen of common carp by chemical and immunochemical methods [19]. In total, the Special Issue comprises 13 articles, including two reviews. It is interesting that six articles are about glycoconjugates from echinoderms and one article concerns the glycoconjugates of a microorganism associated with an echinoderm, i.e., the subject of more than half of the articles, is linked with echinoderms that reveals significant the biomedical potential of this group of marine invertebrates.

In conclusion, the editors are very appreciative to all authors that contributed their excellent works to our Special Issue and wish them new and exciting discoveries.
